# HSP70 mediates survival in apoptotic cells—Boolean network prediction and experimental validation

**DOI:** 10.3389/fncel.2015.00319

**Published:** 2015-08-25

**Authors:** Suhas V. Vasaikar, Sourish Ghosh, Priyam Narain, Anirban Basu, James Gomes

**Affiliations:** ^1^Computational Biology, Kusuma School of Biological Sciences, Indian Institute of Technology DelhiNew Delhi, India; ^2^National Brain Research CentreManesar, India

**Keywords:** neuronal stress, HSP40, HSP70, HSP90, apoptosis, cellular fate, Boolean network

## Abstract

Neuronal stress or injury results in the activation of proteins, which regulate the balance between survival and apoptosis. However, the complex mechanism of cell signaling involving cell death and survival, activated in response to cellular stress is not yet completely understood. To bring more clarity about these mechanisms, a Boolean network was constructed that represented the apoptotic pathway in neuronal cells. FasL and neurotrophic growth factor (NGF) were considered as inputs in the absence and presence of heat shock proteins known to shift the balance toward survival by rescuing pro-apoptotic cells. The probabilities of survival, DNA repair and apoptosis as cellular fates, in the presence of either the growth factor or FasL, revealed a survival bias encoded in the network. Boolean predictions tested by measuring the mRNA level of caspase-3, caspase-8, and BAX in neuronal Neuro2a (N2a) cell line with NGF and FasL as external input, showed positive correlation with the observed experimental results for survival and apoptotic states. It was observed that HSP70 contributed more toward rescuing cells from apoptosis in comparison to HSP27, HSP40, and HSP90. Overexpression of HSP70 in N2a transfected cells showed reversal of cellular fate from FasL-induced apoptosis to survival. Further, the pro-survival role of the proteins BCL2, IAP, cFLIP, and NFκB determined by vertex perturbation analysis was experimentally validated through protein inhibition experiments using EM20-25, Embelin and Wedelolactone, which resulted in 1.27-, 1.26-, and 1.46-fold increase in apoptosis of N2a cells. The existence of a one-to-one correspondence between cellular fates and attractor states shows that Boolean networks may be employed with confidence in qualitative analytical studies of biological networks.

## Introduction

Neurodegenerative diseases are characterized by extensive neuronal apoptosis (Mattson et al., 2000), triggered by various factors responsible for deprivation of survival signals, accumulation of misfolded proteins and reactive oxygen species alteration of Ca2+ homeostasis, elevated calcium levels and mitochondrial dysfunction. For instance, in Alzheimer's disease elevated level of β-Amyloid (Aβ) within the senile plaques (Morishima et al., 2001) resulted in activation of c-Jun N-terminal pathway that leads to selective neuronal death, whereas in Parkinson's disease, mitochondrial dysfunction caused by oxidative stress leads to neuronal apoptosis. (Jenner and Olanow, 1998). In other NDD's like amyotrophic lateral sclerosis oxidative stress causes over-activation of glutamate receptors and overloads cellular calcium thereby causing death of neuron (Culmsee and Krieglstein, 2007). In another study, Sánchez et al. (1999) showed that mutant huntingtin activates the caspase-8 in primary rat neurons and eventually causing cell death thereby manifesting Huntington's disease (Sánchez et al., 1999). Activation of apoptotic factors is central to and responsible for cellular demise via the apoptotic pathway (Figure S1). Since a large number of pro- and anti-apoptotic proteins are involved, understanding the mechanism of neuronal apoptosis is vital to gain insight into the complexity of this pathway across various neurodegenerative diseases.

The apoptosis mechanism is highly complex and regulated at several levels. Certain external stress factors can induce apoptosis through death ligand receptors (Van Herreweghe et al., 2010). For example, FasL(CD95)-receptor interactions induces apoptosis through two major pathways, the extrinsic or death receptor (type I) pathway and the intrinsic or mitochondrial (type II) pathway (Scaffidi et al., 1998; Peter et al., 2007; Bouillet and O'Reilly, 2009). These pathways lead to the activation of caspases and subsequently cell apoptosis. Tyas et al. (2000) and Rehm et al. (2002) have observed that activation of caspase-3 takes hours before the initiation of apoptosis, but once activated they cause rapid cell death, which can be visualized using single-cell imaging techniques. Another study has shown that PC12-D_2_R population of cells undergoing oxidative stress leads to bifurcation of signaling response into two states (survival or death) (Nair et al., 2004). The bi-stability behavior was also derived from a mathematical model for mitochondria-dependent apoptosis, which showed that this was induced by cooperative apoptosome formation (Bagci et al., 2006). These studies demonstrated that a rapid and an all-or-none behavior in apoptosis. These complex multigenic factors involved in manifestation of neurodegenerative disease involving death of neurons makes it essential to develop an *in silico* approach for gaining mechanistic insight into biological pathways involved in disease development and progression.

To gain an insight into to the molecular interaction network that result in opposing outcomes of apoptosis and survival, we constructed a Boolean network integrating various factors associated with different neurodegenerative disorders. Apparently, the architecture of the apoptosis network results in a bimodal response of either death or survival by design. In the resulting Boolean network, each vertex represents a protein and its state is attributed a value of 1, 0, or −1 to indicate if it is activated, unchanged or inhibited, respectively. As time evolves, the network acquires several dynamic states, which eventually reach steady states called “attractors” or “fixed points.” Recent cell cycle studies using Boolean networks have shown that these attractors can be correlated to cell growth, differentiation, and apoptosis (Huang and Ingber, 2000; Albert et al., 2008). These attractors are also associated with network behavior, and it is therefore meaningful to identify them in a biological network. Some studies have also reported the application of Boolean analysis in apoptosis (Huang and Ingber, 2000; Zhang et al., 2008). Mai and Liu (2009) have found that certain attractors from the randomly chosen initial states resulted in the irreversibility of cellular apoptosis (Mai and Liu, 2009). A Boolean model has also been used to study effect of external stimuli such as tumor necrosis factor (TNF) and UV-B irradiation on apoptosis network using different time scales (Schlatter et al., 2009). The introduced time delays described the dynamic processes associated with TNF signaling event. Another report shows how a discrete mathematical modeling may be used to explain the interplay between survival pathway mediated by NFκB, necrosis by RIP1 pathway and apoptosis by death receptor (Calzone et al., 2010).

Several such studies have resulted in a reasonable understanding of numerous intracellular proteins associated with apoptosis due to external stress. Among these, study of HSPs has received a high degree of attention due to their role in the suppression of apoptosis. It has already been well established that HSPs are important molecular chaperones that protect cells from various stresses, and facilitate their recovery (Lanneau et al., 2007). Here, we examined the role of HSPs in mitigating apoptosis by FasL. The members of HSPs superfamily constitute highly conserved and constitutively expressed or induced proteins. They are classified according to their molecular mass that includes high-molecular-mass HSPs (= 100 kD), HSP90 (81–99 kD), HSP70 (65–80 kD), HSP60 (55–64 kD), HSP40 (35–54 kD), and small HSPs (= 34 kD) (Hartl, 1996). A number of studies elucidated that HSPs play anti-apoptotic role in FasL mediated cell death pathway (Beere, 2005). Among these, HSP27 prevents the translocation of BID to the mitochondria, interacts with cytochrome-c, and blocks cytochrome-c mediated interaction between APAF-1 and caspase-9, further inhibiting the caspase-9 activation (Bruey et al., 2000). Similarly, HSP70 and HSP90 are known to interact with APAF-1 and thereby interfere with the type-II caspase activation pathway (Saleh et al., 2000). HSP70 also suppresses the caspase-8 mediated cleavage and activation of BID (Mosser et al., 2000; Clemons et al., 2005). Further, HSP70 and its co-chaperone HSP40 prevented the translocation of BAX to the mitochondria (Gotoh et al., 2004). Therefore, these protective functions of HSPs are indeed significant in reversing the apoptotic states into cell survival states.

Our objective in this study was to identify the critical vertices of the apoptosis network using Boolean analysis and to examine how the balance shifts between apoptosis and survival, how apoptosis caused by stress conditions may be alleviated and verify predictions of network with experiments and reported literature. Since a Boolean network of size *N* requires 2^*N*^ computation steps to determine all the attractors, we were required to enumerate 2^21^ states for our network. The 17 attractors obtained were then grouped into the following cellular fates - survival, apoptosis and DNA repair. From the basin of attractors, the probability of cellular fates was calculated for different initial conditions of input survival factor, growth factor (GF) and FasL. Further, perturbation analysis, where the state of node was fixed at 0 or 1 to represent the genetic equivalent of deletion or overexpression, was performed. The results obtained from vertex perturbations suggested that BCL2, IAP, cFLIP, and NFκB represented critical vertices in the network and acted as checkpoints for apoptosis. In experiments using N2a cell line, inhibition of BCL2, IAP and IKK caused extensive apoptosis. Further, we studied the role of heat shock proteins on cellular fates. The study showed that HSP70 overexpression, in comparison to HSP40 and HSP90, in N2a cells suppressed apoptosis in large number of cells when treated with FasL. The study gives a coherent picture of how cellular fates result from the crosstalk between pro- and anti-apoptotic proteins in a network. An examination of the role of HSPs in the prevention of apoptotic states provides an insight into the transition of a cell from extreme state of apoptosis to extreme state of survival and therefore suggests a probable role of HSP70 for therapeutic purposes in neurodegenerative diseases.

## Material and methods

### Neuronal apoptosis network

**Figure 1:**
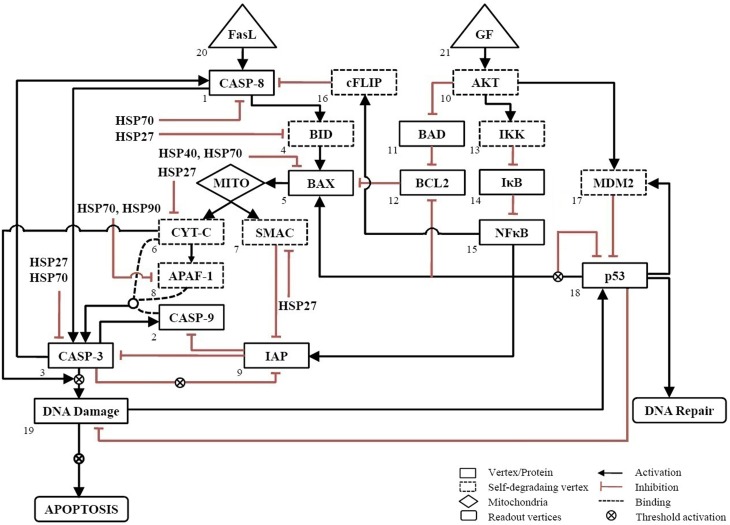
**Neuronal apoptosis network**. The directed network consists of 21 vertices representing proteins and 37 edges describing the regulatory interactions between vertices. The regulatory interactions are shown, wherein activation is represented by → and inhibition by ˧. Self-degrading vertices are shown in dashed-line rectangular box. The input vertices represent survival factor, GF and death ligand, FasL shown in triangular box. The output of network was measured from readout vertices which represent DNA Repair and APOPTOSIS. The activation of Mito box denotes the initiation of mitochondrial death pathway.

The wiring diagram for apoptosis in neuronal cells was constructed based on literature on neuronal and mammalian cells (Nagata, 1999; Mattson, 2000; Poulaki et al., 2001). The network consists of 21 vertices, 37 edges and two inputs, GF and FasL (Figure 1). The input vertex “FasL” activates pro-apoptotic proteins via both pathway type I and II. The “GF” input signal in the network activates the anti-apoptotic proteins, which regulate the expression of pro-apoptotic proteins. In addition, the regulations of pro-apoptotic molecules by heat shock proteins HSP27, HSP40, HSP70, and HSP90 have been included in the network to study the mitigating effects of these proteins on apoptosis (Bruey et al., 2000; Mosser et al., 2000; Saleh et al., 2000; Gotoh et al., 2004; Beere, 2005; Clemons et al., 2005).

Each vertex can have one of the two possible values as either 1(ON) or 0(OFF) at any given point of time. The edges are directed and carry a (+) sign for activating edges and a (–) sign for inhibiting edges. From any given initial state of the network, the subsequent states were determined by the threshold function defined as below,

(1) $$\begin{array}{l} S_{i}(t+1)=1\;if\;\sum a_{ij}S_{j}(t)>0\\ S_{i}(t+1)=0\;if\;\sum a_{ij}S_{j}(t)<0\\ S_{i}(t+1)=S_{i}(t)\;if\;\sum a_{ij}S_{j}(t)=0 \end{array}$$

where *S*_*i*_(*t*+1) represents state of vertex at time *t* + 1. At any vertex, α_*ij*_ = +1 for an activating input and −1 for a inhibiting input. The vertices, which were not regulated in the network, were considered as self-degrading vertices (Albert et al., 2008). For self-degrading vertices the threshold function given as follow

(2) $$S_{i}(t+1)=0\;if\begin{array}{c} \sum a_{ij}S_{j}(t)\leq 0 \end{array}$$

We call a consecutive sequence of outcomes for the *i*^*th*^ vertex denoted by *v*_*i*_(*t*), *v*_*i*_(*t*+1), …, *v*_*i*_(*t*+*p*) an *attractor* with period *p* if *v*_*i*_(*t*) = *v*_*i*_(*t*+*p*). For each initial state, an attractor state was obtained after synchronous updating algorithm simulated in MATLAB. The number of computation steps was 15 and it equaled the longest path length for this network.

In addition to the generalized rules [Equations (1) and (2)], some constraints were added to account for specials conditions at certain vertices. To mimic the threshold activation of DNA damage when caspase-3 and cytochrome-c are amplified (Deshmukh et al., 2000; Chang et al., 2003), the output from this vertex was triggered if it was ON for three successive time steps otherwise not. Since IAPs are inhibited when caspase-3 is amplified, it was triggered only when caspase-3 remained ON for two successive time steps (Albeck et al., 2008). Similarly, because the amplification of p53 up-regulates expression of BAX, down-regulates expression of BCL2 protein and inhibit its own expression (Kim et al., 1998; Karpinich et al., 2002; Jõers et al., 2004), was triggered only the vertex was ON for two successive time steps.

To simulate a loss-of-function mutation, the value of vertex was set to 0 (OFF) to remove its interactions with other vertices. A gain-of-function or over-expression was simulated by fixing the value of the vertex to 1 (ON). The attractors were correlated to cell fate by categorizing these into survival, apoptosis, and DNA repair. The outcome of the network resulting from a combination of GF and FasL was obtained as “DNA Repair” or “Apoptosis.” The relationship used to construct the network is summarized in Table S1. The nomenclature of all vertices of the network has been provided in the Table of abbreviations.

### Cell culture and assessment of cell viability

Mouse neuroblastoma cell line (N2a) was cultured in Dulbecco's minimal essential medium (DMEM) supplemented with 10% heat-inactivated fetal bovine serum (FBS) (Gibco), 100 U/ml penicillin and 100 μg/ml streptomycin in a standard humidified incubator at 37°C and 5% CO_2_. Cells were seeded in chamber slides with cell density 2 × 10^4^ – 5 × 10^4^ cells/cm^2^. Cells were treated with Nerve Growth Factor (NGF, 2 nM) and FasL (2 nM), appropriate chemical inhibitors and then incubated for overnight at 37°C. The cell viability was assessed using CellTiter 96 Proliferation Assay kit (Promega, USA). After 12 h of treatment, 20 μL of MTS solution was added in each well. It was quantified by measuring the light absorbance after 4 h of incubation at 450 nm in an ELISA plate reader (Multiskan-GO, Thermo Scientific, MA, USA). The background absorbance was measured at 550 nm in wells containing MTS solution and culture media. The results were expressed as the percent change in optical density (OD) values for treated samples and untreated control wells.

### Transfection of heat shock protein plasmids

The plasmids pcDNA5-HSP40 (Plasmid19495), pEGFP-HSP70 (Plasmid15215), and pcDNA3-HSP90 (Plasmid22487) from Addgene (Addgene, USA) were used for N2a transfection. The GFP reporter gene was present in plasmids pcDNA5-HSP40 and pEGFP-HSP70, and HA reporter gene in pcDNA3-HSP90. The N2a cells were grown in 24 well plate containing FBS and antibiotics. Prior to transfection the growth media was replaced with serum free media, followed by transfection of 10 μM plasmids using Lipofectamine RNAi max (Invitrogen, Carlsbad). The cells were then treated with FasL for 12 h and subsequently TUNEL assay was performed.

### Inhibitors treatment

BCL2 Inhibitor III (EM20-25, 5-(6-chloro-2,4-dioxo-1,3,4,10-tetrahydro-2H-9-oxa-1,3- diaza-anthracen-10-yl)-pyrimidine-2,4,6-trione), IKK inhibitor (Wedelolactone), and IAP inhibitor (Embelin) (Calbiochem, San Diego, CA) were dissolved in dimethyl sulfoxide to form a stock concentration of 1 mM. The stock was aliquoted and stored at −20°C. The inhibitor molecules were diluted to a working concentration of 10 μM using DMEM medium. For each experiment fresh aliquot of inhibitors was used and 10 μM added in each well. After 4 h of incubation, cells were treated with FasL (2 nM). The cell death was examined after 12 h of incubation by TUNEL assay.

### Quantitative RT PCR

Cells treated with inhibitors were lysed with Trizol reagent (Invitrogen Life Technologies, Carlsbad, CA) for total RNA isolation. Subsequently, RNA extract was treated with TURBO DNA-free™ Kit (Ambion, Carlsbad, USA) in order to get rid of DNA contamination. The treated samples were thereafter subjected to cDNA synthesis using random hexamers (Amersham) at 65°C for 10 min and then at 37°C for 1 h. Quantitative RT-PCR was performed for gene specific transcript using primers specific for mouse were: caspase-8-Fwd, 5′-GAGGATGCCCAGAAGCTA-3′; caspase-8-Rev, 5′-CCG CAGCTCTCTCACCAT-3; caspase-3-Fwd, 5′-AATGTCATCTCGCTC TTGGT-3′; caspase-3-Rev, 5′-GCTTAGAATCACAC ACAC-3′; BAX-Fwd, 5′-CACCAGCTCTGAACAGAT-3′; BAX-Rev, 5′-CCAGTTCATCTCCAATTCG-3′. The reactions were carried out in an ABI Prism 7500 sequence detection system (Applied Biosystems, CA, USA). The qRT-PCR results were analyzed using the iCycler Thermal Cycler Software (Applied Biosystems, CA, USA) and normalized with those from 18S rRNA internal control (Das et al., 2009).

### Terminal deoxynucleotide transferase-mediated dUTP nick-end labeling (TUNEL) assay

N2a cells were plated at a density of 5 × 10^4^ cells/well on eight-well chamber slides (Nunc, Denmark) and were analyzed for cell death analysis using Cell Death Detection Kit, TMR red (Roche, Germany). The cells were fixed with 4% paraformaldehyde in PBS and blocked with 4% BSA containing 0.02% Triton-X-100. The fixed cells were then incubated in TUNEL mix (terminal deoxynucleotidyl transferase) in storage buffer and TMR red labeled-nucleotide mixture in reaction buffer) for 1 h at room temperature as per the manufacturer's protocol. The slides were mounted with Vectashield mounting media containing 4′-6-diamidino-2-phenylindole (DAPI; Vector Laboratories, USA) and observed under a Zeiss Axioplan 2 upright digital microscope.

### Statistical analysis

The statistical analysis was performed using the Student's *t*−test. *P*−values that were < 0.05 were considered statistically significant. Average values were expressed as mean ± standard deviation.

## Results

### Extreme cases in apoptosis network

The neuronal apoptosis network was constructed from literature studies as shown in Figure 1. Boolean analysis of 2^21^ initial states of this network led to the identification of 17 singleton attractors. These attractors depict the steady state at which the expression of each protein in the network is represented as active (1) or inactive (0). For example, the attractor 1 obtained when GF and FasL were turned OFF is presented as “111111110010010011100.” Here, binary value for each protein in the network in the order of CASP8 (caspase-8), CASP9 (caspase-9), CASP3 (caspase-3), BID, BAX, CYT-C (cytocrome-c), SMAC, APAF, IAP, AKT, BAD, BCL2, IKK, IκB, NFκB, cFLIP, MDM2, p53, DNA damage, FasL, and GF, respectively shows if it is active (1) or inactive (0) (Table 1). These attractors represent steady states and are correlated to the cellular fates resulting from the initial conditions. Out of 17 attractors, 9 attractors were stable throughout 15 steps of one cycle of simulation and the remaining 8 attractors were stable initially but transited to a different state after 6–7 steps and are called transient attractors (Table 1). The 17 attractors were categorized into three cellular fates–survival (9 attractors), apoptosis (6 attractors), and DNA repair (2 attractors). The probability of each cellular fate was calculated for different initial conditions of FasL and GF in a network. The ON state of GF and OFF state of FasL results into seven attractors (attractor 2, 3, 10, 11, 12, 13, 14; Table 1). The probability of survival fate (*f* = 0.60) and apoptosis (*f* = 0.40) was observed. Among seven attractors, 59% of initial states converged to attractor 2 (Table 1). For attractor 2, anti-apoptotic proteins cFLIP, BCL2, IAP, AKT, NFκB, MDM2, and IKK were “ON.” This result suggests that the ON state of anti-apoptotic molecules in the network represented the most stable state. In presence of FasL, the initial conditions converged to single attractor (attractor 4) in which anti-apoptotic proteins were in OFF state and pro-apoptotic proteins were in ON state. Thus, the activation of FasL results in a single attractor that corresponds to apoptosis. In presence of both FasL and GF, initial conditions converged to eight attractors (attractor 5, 6, 7, 8, 9, 15, 16, 17; Table 1). The probability of apoptosis (*f* = 0.96) was higher compared to survival (*f* = 0.016), and DNA repair (*f* = 0.024).

**Table 1:** **Attractors obtained from Boolean analysis of apoptosis network**.

**Attractor no**.	**Attractor basin**	**Initial condition**		**Attractor**	**Cell fate**	**Stability**
		**GF**	**FasL**			
1	524288	OFF	OFF	“111111110010010011100”	Cell death	Stable
2	309976	ON	OFF	“000000001101101110001”	Survival	Stable
3	186784	ON	OFF	“111111110101101111101”	Cell death	Stable
4	524288	OFF	ON	“111111110010010011110”	Cell death	Stable
5	476871	ON	ON	“111111110101101111111”	Cell death	Stable
6	5117	ON	ON	“100100001101101110011”	Survival	Stable
7	11859	ON	ON	“100111111101101111111”	DNA repair	Stable
8	3482	ON	ON	“000000001101101110011”	Survival	Stable
9	621	ON	ON	“110111111101101111111”	DNA repair	Stable
10	23520	ON	OFF	“111111110100101111101”	Cell death	Transient
11	816	ON	OFF	“011011110100101111101”	Survival	Transient
12	2712	ON	OFF	“011011110101101111101”	Survival	Transient
13	336	ON	OFF	“000011111101101111101”	Survival	Transient
14	144	ON	OFF	“010011111101101111101”	Survival	Transient
15	48	ON	ON	“000011111101101111111”	Survival	Transient
16	18	ON	ON	“010011111101101111111”	Survival	Transient
17	26272	ON	ON	“111111110100101111111”	Cell death	Transient

**Figure 2:**
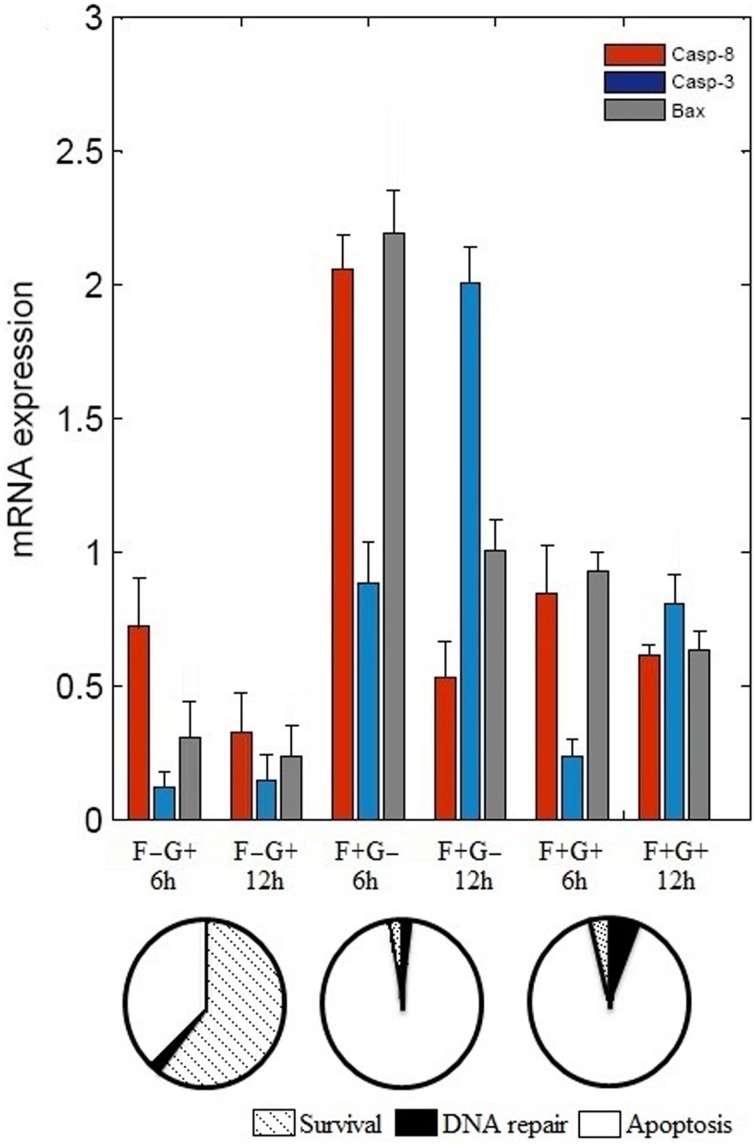
**Effect of FasL and NGF on N2a cells viability**. Cells were incubated with NGF (2 nM) and FasL (2 nM) for 12 h in combinations such as, (i) NGF (F–G+), (ii) FasL (F+G–), (iii) FasL and NGF (F+G+), and (iv) maintenance media (F–G–) which acts as a control. Change in mRNA level profile of the caspase 8, caspase 3, and BAX was measured after 6 h and 12 h of incubation by real-time PCR (mean ± SD, *n* = 3). Probabilities of cellular fates observed from attractors are shown in the pie chart corresponding to initial conditions of GF and FasL.

The binary values of 17 attractors were examined and frequency of “ON” state of each vertex was calculated. To test the frequency of “ON” state predictions, we examined mRNA level of caspase-3, caspase-8, and BAX by reverse transcriptase polymerase chain reaction (RT-PCR) (Figure 2). N2a cells were grown in four different conditions that are presence of (i) NGF, (ii) FasL, (iii) NGF and FasL, and (iv) maintenance media. After 12 h of incubation with NGF, mRNA level of caspase-8, caspase-3, and BAX was increased by 0.33, 0.14 and 0.23 times, respectively. After FasL treatment, mRNA level of caspase-3 increased by 2 times whereas for caspase-8 and BAX mRNA level increased by 0.5 and 1 times, respectively. Addition of both NGF and FasL showed more than 0.6 times increase in caspase-8 and BAX mRNA level and 0.8 times increased caspase-3 mRNA level. Increased expression of caspase-3 and caspase-8 is indicator of CD95-mediated apoptosis [8]. This result demonstrates the correlation with frequency of “ON” state predictions results obtained from Boolean analysis. In absence of GF and FasL, Boolean analysis predicted apoptosis fate of attractor for network. However, in absence of nerve growth factor, N2a cells do not show significant increase in apoptosis over period of 24 h but increased rapidly after 40 h (data not shown).

### HSP70 protects cells against fasL induced apoptosis

**Figure 3:**
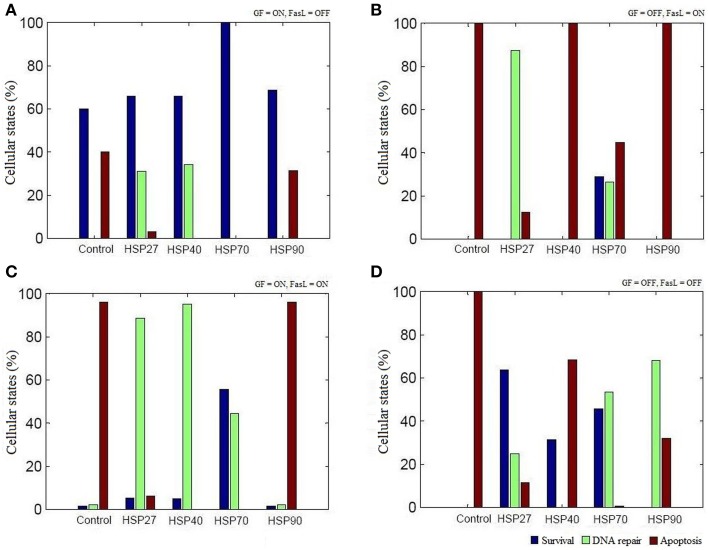
**The role of HSPs in apoptosis network**. The Boolean analysis was performed using HSP27, HSP40, HSP70, and HSP90 regulation in apoptotic network. The attractors were obtained by performing simulations under four different combinations of GF and FasL. **(A)** ON state of GF showed maximum states were in survival fate. The active state of HSP70 resulted in significantly increase in survival fate. **(B)** When FasL was active and GF was inactive, ON state of HSP27 and HSP70 resulted in reduced apoptosis states. **(C)** In the presence of both GF and FasL, more than 40% of states were in DNA repair fate and showed decreased apoptosis states in HSP27, HSP40, and HSP 70. The ON state of HSP70 showed more than 50% of states having survival fate. **(D)** When GF and FasL were in OFF state, HSP27and HSP70 showed more than 40% of states in survival states.

The regulation of various HSPs involved in apoptosis was curated from the literature and assigned Boolean functions (Figure 1). To study the role of HSPs (HSP27, HSP40, HSP70, and HSP90) in the apoptosis network, we performed Boolean simulation under four different initial conditions of GF and FasL. The Boolean attractors obtained from these simulations suggests that when GF was active (ON), high probability of survival states was observed in all HSPs and the probability of survival fate was maximum in HSP70 (Figure 3A). The probability of survival fate observed in HSP27 and HSP40 was comparable with wild type network (apoptosis network without HSPs). When FasL was active (ON), it resulted in a 28.8% increase in the number of survival states in HSP70 and 87.5% increased DNA repair states in HSP27 (Figure 3B). The active (ON) state of HSP40 and HSP90 does not result in a change in probability of apoptosis. When GF and FasL were ON, HSP27, HSP40, HSP70, and HSP90 resulted in 5.4, 4.9, 55.6, and 1.7% survival states, respectively (Figure 3C). When the effect of regulation of HSP27 and HSP70 on the apoptosis network was considered in the absence of GF and FasL, 40% of the states resulted in cell survival (Figure 3D). The overall results obtained from Boolean analysis of the apoptosis network, with the HSP regulation included, showed that when HSP70 was active (ON) in the network the probability of survival was increased by ~25%. Further we observed that when FasL was ON, the active state of HSP70 reduced the number of apoptotic states by 60% compared to the results obtained in the absence of HSP regulation.

**Figure 4:**
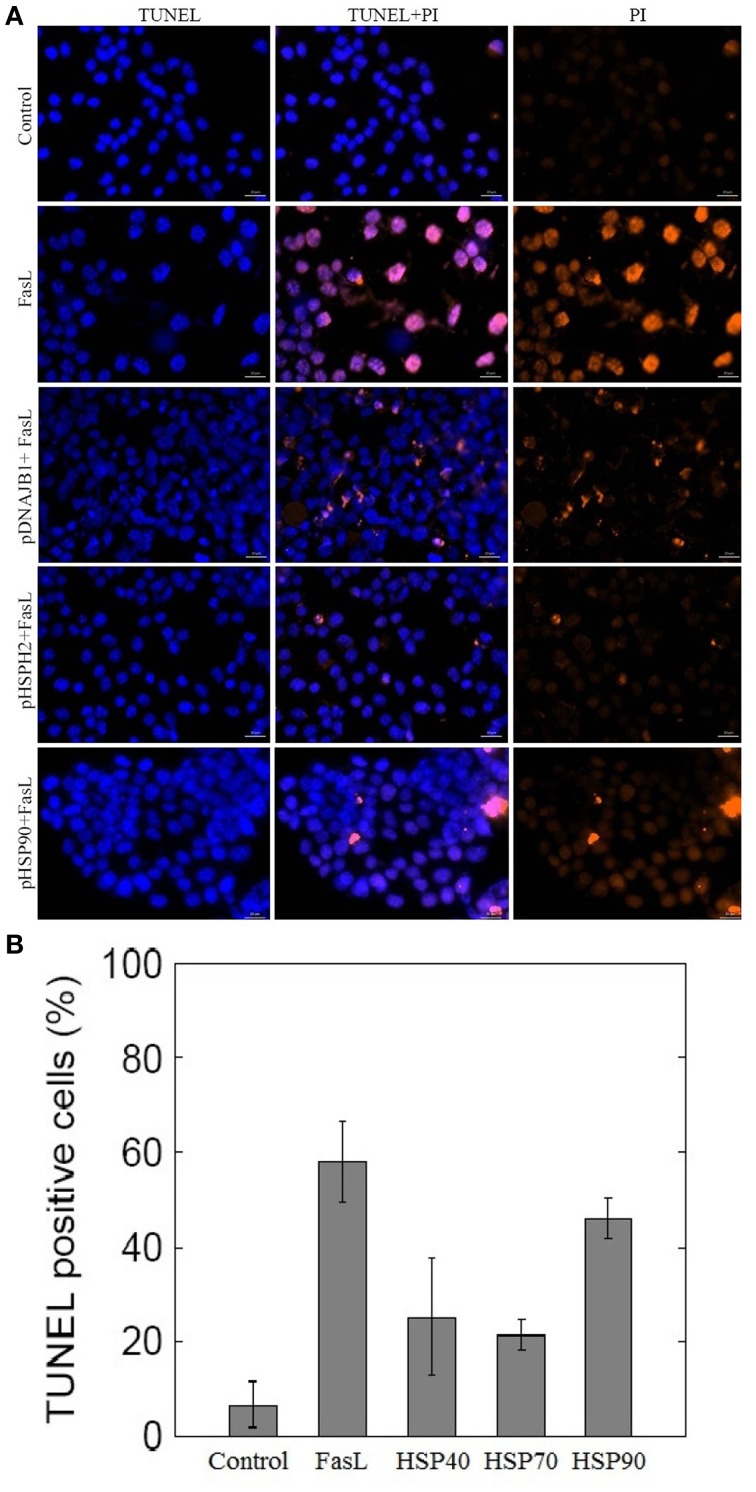
**Effect of overexpression of HSPs on FasL-induced apoptosis in N2a cells**. **(A)** N2a cells were transfected with HSP40, HSP70, and HSP90 gene. The transfected cells were treated with FasL and apoptotic cells were measured after 12 h of incubation. The apoptosis of N2a cells were determined using TUNEL assay. **(B)** The bar graph displays the mean ± SD (Standard deviation) percentage of apoptosis measured from TUNEL positive cells.

To investigate whether HSPs could suppress FasL induced apoptosis in N2a cells, we transfected N2a cells with plasmids containing HSP40 (DNAB1), HSP70 (HSPH2), and HSP90 gene (Material and Methods). The ability of N2a cells to inhibit apoptosis was examined by employing TUNEL assay. As shown in Figures 4A,B, treatment of HSP transfected N2a cells with FasL for 12 h decreased the number of TUNEL positive apoptotic nuclei when compared with wild type of N2a cells treated with FasL. The expression of HSP40 and HSP70 gene in transfected cells suppressed apoptosis by almost 2.5 times (Figure 4B). HSP90 gene transfected cells did not show significant change in the number of apoptotic cells.

### Critical vertices in apoptosis network

**Figure 5:**
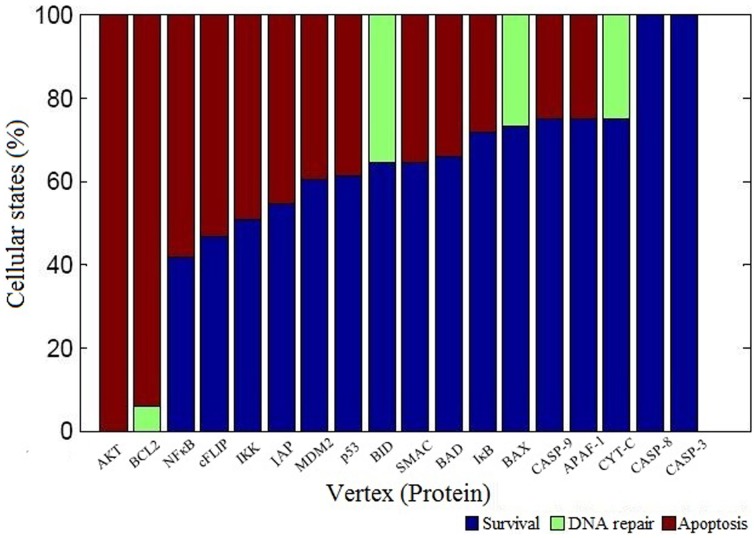
**Vertex perturbation in apoptosis network**. The graphical representation of cellular fates obtained after perturbation of node is shown. The OFF state of anti-apoptotic nodes such as AKT, BCL2, NFκB cFLIP, and IKK resulted in significant apoptosis states (red color). However, perturbation of pro-apoptotic nodes such as CASP3 and CASP8 resulted in maximum survival states (blue color). In case of BCL2, BID, BAX, and CYT-C mutation, few states were observed in DNA repair states shown in light green color.

We performed vertex perturbation analysis to examine the critical pro- and anti-apoptotic proteins in apoptosis network. The OFF state of anti-apoptotic proteins leads to a decrease in probability of survival fate as seen from mutations in AKT (*f* = 0), BCL2 (*f* = 0), NFκB (*f* = 0.42), cFLIP (*f* = 0.47), IKK (*f* = 0.51), and IAP (*f* = 0.55) illustrated in Figure 5. When BCL2 was computationally mutated in the network, it resulted in higher fraction of apoptotic cells suggesting that key role of BCL2 protein in cell survival. On the other hand, OFF regulation of pro-apoptotic proteins such as caspase-3 (*f* = 1), caspase-8 (*f* = 1), and caspase-9 (*f* = 0.75) showed increased survival states. When the mitochondrial factors such as CYT-C (*f* = 0.75), APAF (*f* = 0.75), BAX (*f* = 0.73), SMAC (*f* = 0.65), and BID (*f* = 0.65) were turned off by mutation, the probability of survival fate was increased.

**Figure 6:**
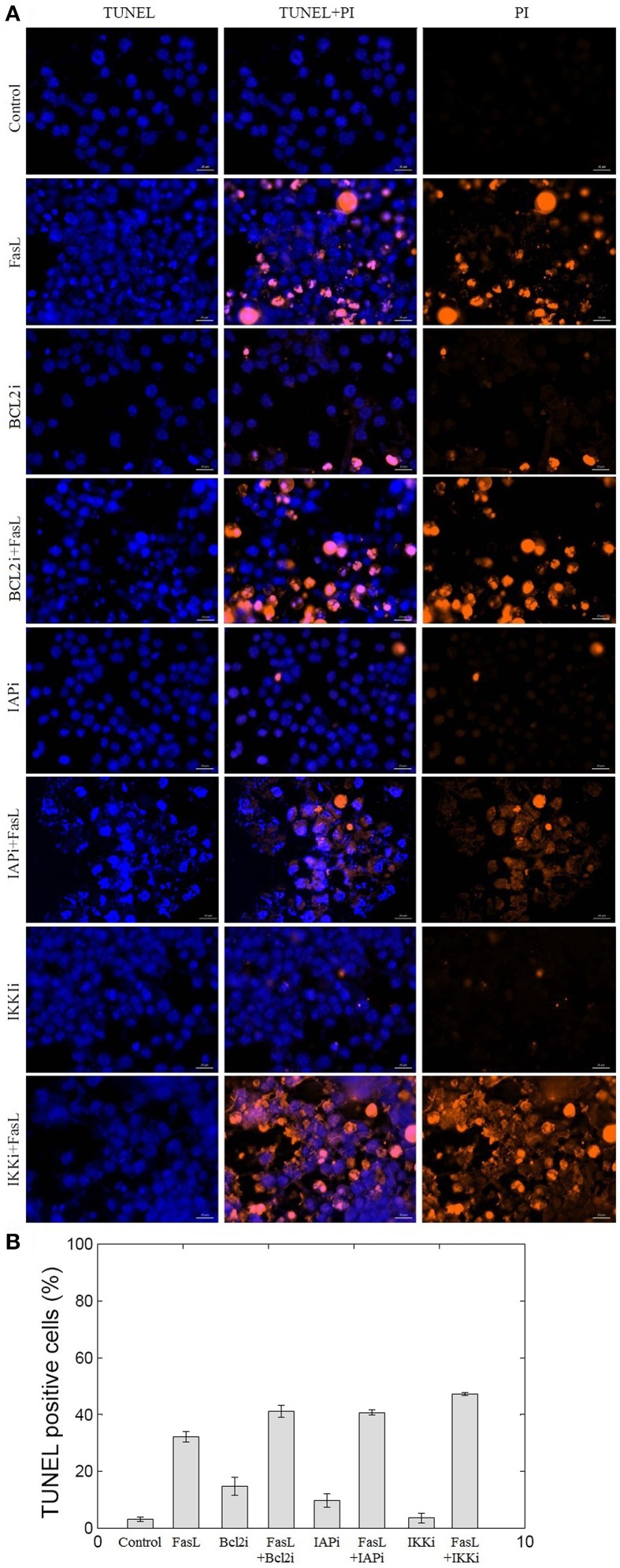
**Effect of BCL2, IAP, and IKK inhibitors on N2a cell viability**. The inhibition of BCL2, IAP, and IKK proteins was performed using EM20-25, Embelin and Wedelolactone, respectively. **(A)** The apoptosis of N2a cells were determined using TUNEL assay. **(B)** The bar graph displays the mean ± SD (Standard deviation) ratio of apoptosis after 12 h of incubation. EM20-25 caused inhibition of BCL2 protein that resulted in increased cell death after FasL treatment. Similarly, treatment of cells with Embelin and Wedelolactone resulted in increased TUNEL positive cells. The following concentrations were used: EM20-25, 10 μM/ml; Embelin, 10 μM/ml; and Wedelolactone, 10 μM/ml.

To experimentally validate the results obtained from the perturbation of anti-apoptotic proteins in the network, we studied inhibition of BCL2, IAP, and IKK proteins in N2a cells. BCL2 and IAP are key regulators of mitochondrial apoptosis pathway and caspase cascade, respectively. IKK regulates NFκB, which in turn regulates the expression of anti-apoptotic proteins. Hence, to study the role of these proteins in neuronal apoptosis network, N2a cells were treated with EM20-25 (BCL2 inhibitor), Embelin (IAP inhibitor), and Wedelolactone (IKK Inhibitor II) independently at 10 μM of working concentration. Apoptosis in N2a cell was induced by FasL (Figure 6). The nuclear morphology of apoptotic cells was examined with a fluorescent DNA-binding agent (TUNEL staining). The FasL treatment of N2a cells resulted in 32.2% TUNEL positive cells which was 10-fold of control. The EM20-25 treatment resulted in 14.7% TUNEL positive cells but further treatment with FasL caused increased apoptosis in N2a cells (41.12%). Similarly, 9.6 and 3.6% apoptotic cells were observed when cells were treated with Embelin and Wedelolactone, respectively. However, pre-treatment with Embelin and Wedelolactone followed by FasL addition into media resulted in 40.7 and 47.2% apoptotic cells, respectively. Taken together, these results suggested inhibition of BCL2, IAP, and IKK by EM20-25, Embelin and Wedelolactone, resulted in 1.27−, 1.26−, and 1.46− fold increase respectively in apoptosis in N2a cells. Thus, BCL2, IAP, and IKK seem to be the critical proteins essential for survival of N2a cells.

## Discussion

### Cellular fates

In this study, we constructed a neuronal apoptosis network that represents Boolean graph of size *G* = {21, 37} as shown in Figure 1. The computations were carried out using Boolean analysis to identify the cellular fates and underlying key regulators. The computation resulted in 17 attractors from initial state space (2^21^) thus representing cellular fates which were grouped into survival, apoptosis, and DNA repair (Table 1). The probability that the initial state would move toward one of these three fates, in the presence of two input factors GF and FasL, was calculated from the basin of attractors. In the presence of GF, the probability of survival (*f* = 0.60) was higher than apoptosis (*f* = 0.40). The attractors showed that anti-apoptotic molecules were ON which includes cFLIP, BCL2, IAP, AKT, NFκB, MDM2, and IKK proteins. The frequency of ON state of vertices was calculated in survival fate that showed high frequency of activation of cFLIP (59.89), BCL2 (59.73), IAP (59.21), NFκB (59.89) proteins, whereas relatively lower frequency was observed in caspase-8 (0), caspase-3 (0.67), BAX (0.76), and CYT-C (0.76). The mRNA level of caspase-8, caspase-3, and BAX was measured by RT-PCR showed 0.33, 0.14, and 0.23 times increase, respectively (Figure 2). These results suggest that only a few cells showed activation of caspases and hence initiation of apoptosis. Similarly, the cellular fates in the presence of FasL and in the absence of GF were studied. It was observed that all the states moved to single attractor (attractor 4, Table 1) representing apoptosis fate. This attractor (attractor 4, Table 1) showed significant increase in the frequency of ON state of pro-apoptotic proteins such as caspase-8, caspase-9, caspase-3, BID, and BAX. The mRNA level of caspase-8, caspase-3, and BAX was increased by 0.5, 2, and 1 times, respectively (Figure 2). The results showed increased expression level of caspase-3, suggesting initiation of apoptosis significantly. Further when both GF and FasL were ON, the eight attractors were obtained whose probability for apoptosis (*f* = 0.96) was higher than survival (*f* = 0.016) and DNA repair (*f* = 0.024). It showed significantly high frequency of ON state of caspase-8, caspase-3, and BAX. The mRNA expression analysis showed 0.6 times increase in caspase-8 and BAX expression level and 0.8 times increase in caspase-3 expression level. The high frequency of ON state of pro-apoptotic proteins was observed in Boolean network. This might be because the attractors were obtained from 2^*N*^ initial conditions after synchronous update which accounts for total possible states that the network can traverse. Therefore, the attractors predicted from the Boolean network are relatively closer to the real network as observed from mRNA expression profiles of caspase-3, caspase-8, and BAX genes.

### Protective role of HSP70 in neuronal apoptosis

We examined the role of HSP27, HSP40, HSP70, and HSP90 in an apoptosis network by Boolean analysis (Figure 1). The presence of HSPs in the apoptosis network resulted in a reduction of apoptosis states. Initial condition with ON state of GF in a network, resulted in a high probability of states in survival fate (*f* = 0.6) and presence of HSPs have increased the probability of survival states compared with network without HSPs (Figure 3A). Among HSPs, HSP70 regulation in the network resulted in significant increase in survival fate states. In presence of FasL, HSP70 showed low probability of states in survival fate (*f* = 0.29) and HSP27 resulted high probability of states in DNA repair fate (*f* = 0.87) (Figure 3B). However, the network without HSPs showed a single apoptotic fate. Similarly, HSP70 resulted in significant number of survival states in presence of both NGF and FasL compared to other HSPs (Figure 3C). When GF and FasL were inactive (OFF), network showed significant apoptotic states whereas HSP27, HSP40, and HSP70 regulation caused increase in survival fate (Figure 3D). The presence of HSP90 regulation in a network resulted in significant states (67.91%) in DNA repair fate. Therefore, decrease in the probability of apoptosis states suggests that presence of HSPs in a network cause reversal of extreme apoptotic state to extreme survival state. Further, the data suggests that caspase amplification and mitochondria mediated signaling resulting in a commitment to cell death, was suppressed by HSP27 and HSP70; hence, these are key proteins that prevent apoptotic states.

To test the role of HSPs in suppression of apoptosis, N2a cells were transfected with plasmids containing HSP40, HSP70, and HSP90 gene. Overexpression of HSP40, HSP70, and HSP90 gene in N2a cells reduced the number of FasL induced apoptosis (Figures 4A,B). Among the examined HSPs, HSP70 was observed to suppress apoptosis significantly in N2a cells (2.7-fold). Similarly, the low expression levels of HSP70 in the ME–180 human cervical carcinoma cells resulted in reduced number of survival cells following treatments with tumor necrosis factor (Jäättelä et al., 1998). Further, the study showed that the overexpression of HSP70 protected ME-180 cells from caspase-3-induced cell death. In another study, overexpression of HSP70 in U937 cells prevented the activation of caspase-3 and DNA fragmentation when exposed to lethal heat shock treatment (Li et al., 2000). Thus, our study reports that HSP70 overexpression correlates with reduced number of apoptosis in N2a cells.

### Key regulators in apoptosis network

The perturbation of vertices was carried out to determine the key regulators in the network. The OFF state of anti-apoptotic proteins such as AKT, BCL2, NFκB, cFLIP, IKK, and IAP showed significant decrease in probability of states in survival fate (Figure 5). It was observed that apart from AKT, BCL2 vertex inactive state (OFF) resulted in significant number of apoptotic states. This indicates that in a network, BCL2 has an important role in regulation of apoptotic pathway by inhibiting caspase dependent and independent pathway. It was reported that inhibition of BCL2 protein affected the regulation of pro-apoptotic proteins thus acting as a major control point for cell death (Danial and Korsmeyer, 2004). Similar observations were made in *Caenorhabditis elegans* where expression of *ced-9* (BCL2 homolog) is required for preventing cell death (Hengartner and Horvitz, 1994). Under OFF state of caspase-3, caspase-8, and caspase-9, significant decrease in probability of apoptosis fate was observed. Thereby, these results suggest that caspases were critical vertices for promoting apoptosis. Cellular knockout studies showed that inhibition of expression of these caspases suppresses apoptosis pathway (Kuida et al., 1998; Porter and Jänicke, 1999; Kruidering and Evan, 2000). Among mitochondrial death pathway proteins, OFF regulation of BID and CYT-C vertex resulted in decreased ON state of caspase-3 and number of apoptotic states (Table 1). Thus, inhibition of BID and CYT-C were most promising target for preventing caspase amplification. Yin et al. (1999) observed that about half of BID^−∕−^ mice showed resistance toward hepatic cell apoptosis when treated with Fas antibodies and also expression of caspase-3 was not detected (Yin et al., 1999). Similarly when superior cervical ganglion neurons were treated with antibodies against cytochrome-c, it suppressed the apoptosis in neurons induced by NGF withdrawal (Neame et al., 1998). Another key regulator of apoptosis observed was cFLIP, it plays a significant role by acting as initial level of defense. It inhibits caspase-8 activation by death ligand (attractor 2, 11, 12, 13, 14, Table 1). It was observed that activation of caspase-8 is a stage-limiting step in extrinsic apoptosis and cFLIP is the major factor that regulates its activation (Schleich and Lavrik, 2013). Poulaki et al. (2001) observed that downregulation of cFLIP had a moderate effect on Fas-mediated apoptosis but both cFLIP and BCL2 down regulation can reverse the Fas resistant state of neuroblastoma. Thus, Boolean network predicted inhibition of cFLIP suggests its key role in survival and similar results were observed from published literature. Another, control point was observed through vertex mutation and attractor analysis of IAP protein. Inhibition of IAP resulted in decrease in probability of survival fate (*f* = 0.55). Also, the frequency of ON state of IAP in observed attractors showed that they inhibited the amplification of the pro-apoptotic proteins in the caspase cascade (state 3, 5, 7, 9, 13, 14, Table 1). Similar results were suggested in study of expression of caspases on sympathetic neurons. The NGF withdrawal of sympathetic neurons causes release of cytochrome-c but does not cause cell death until the IAPs are degraded (Deshmukh et al., 2000). In Drosophila, IAPs (DIAPs) lowered the threshold of caspase activation, which suggests their role as checkpoint in apoptosis (Orme and Meier, 2009). These results showed a correlation between Boolean vertex perturbation results and reported observations in literature.

We tested the effect of inhibition of anti-apoptotic proteins BCL2, IAP, and IKK on N2a neuronal cells. To examine the effect of EM20-25 on neuronal cells, N2a cells were treated with EM20-25 only and with EM20-25 for 2 h followed by FasL for 12 h. This resulted into induced apoptosis 14.7% (N2a cells treated with EM20-25) and 41.1% (N2a cells treated with EM20-25 and FasL) as observed from TUNEL assay (Figure 6). Milanesi et al. (2006) observed that when PC3 cells were treated with EM20-25, cytotoxic effect by binding to BCL2 protein results. This binding disrupts the regulation of BCL2 toward pro-apoptotic proteins, BAX and BAK (Brenner and Mak, 2009; Chipuk et al., 2010). Thus, inhibition of BCL2 protein results in significantly increased probability of apoptosis fate in network as well as in N2a cells. Inhibition of IAP protein and IKK protein by Embelin and Wedelolactone caused reduction in cell survival by 1.26− and 1.46− fold, respectively. Inhibition of IKK resulted in 47.2% of cells TUNEL positive. IKK regulates the NFκB activation and thus control the expression of several cellular proteins involved in immune response, cell proliferation and cell survival (Mattson and Camandola, 2001). However, Figure 5 depicts that the mutation in IKK leads to apoptosis in ~50% of states. This could be due to interactions of IKK regulated NFκB with other cellular proteins that are not considered in the model.

## Conclusion

In this study, we examined the effect of HSPs on apoptosis network through Boolean analysis and validated these findings on N2a cells in presence of external death inducing factor FasL. HSP70 transfected N2a cells suppress apoptosis significantly compared to that observed in control, HSP40 and HSP90 overexpressing N2a cells. The results showed that HSP70 plays an important role in apoptosis by suppressing apoptosis pathway significantly and protecting the cells from FasL-induced death. The study also focused on the interplay between pro- and anti-apoptotic molecules deciding cellular fate decision. The Boolean analysis of network resulted in 17 attractors. These attractors showed that the initial conditions of GF and FasL lead to three cellular fates - survival, DNA repair and apoptosis. To identify the critical proteins in the network, vertex mutation was carried out which showed that BCL2, IAP, cFLIP, and NFκB are critical proteins for cell survival and act as cell death barriers. The predictions were tested by performing inhibition of BCL2, IAP and IKK proteins in N2a cells which showed an increase in apoptosis by 1.27−, 1.26−, and 1.46-fold, respectively. Further, it was observed that inhibition of BID and cytochrome-c were most promising targets for preventing caspases activation. Overall, Boolean apoptosis network depicted a key role of HSP70 in suppressing apoptosis and identified cell death barriers for cell survival. Thus, the Boolean network model formulated in this study can complement experimental studies in understanding the apoptotic process and survival states stability.

### Conflict of interest statement

The authors declare that the research was conducted in the absence of any commercial or financial relationships that could be construed as a potential conflict of interest.
