# Supplementary material for: HSP70 mediates survival in apoptotic cells—Boolean network prediction and experimental validation
Source: Front Cell Neurosci. 2015 Aug 25;9:319. doi: 10.3389/fncel.2015.00319 (PMC4548197; doi:10.3389/fncel.2015.00319)
Supplement: Supplementary file 1 [file Table1.DOC]

**Supplementary Figure**

**Figure S1. Neuronal cell death signaling in neurodegenerative diseases.** In Alzheimer's disease, elevated levels of Aβ induced apoptosis through c-Jun N-terminal kinase (JNK) pathway, leads to expression of Fas ligand. Fas ligand in turn then initiates a cascade of events leading to cell death (Morishima *et al*., 2001). Deregulation of calcium was also observed in AD (Su *et al*.,2003; Tu *et al*.,2006). In Parkinson's disease, increased oxidative stress, mutation in genes like α-synuclein caused mitochondrial dysfunction (Mattson *et al*., 2000; Jenner and Olanow, 1998). Mutant huntingtin found in Huntington's disease was involved in activation of caspase-8 in cultured cells and resulted into caspase-8 mediated apoptosis (Sánchez *et al*., 1999; Polymeropoulos, 1998). Ischemic neuronal death was mediated by an increase in the levels of intracellular calcium level and reactive oxygen species (Wellington *et al*., 2000). Amyotrophic lateral sclerosis is characterized by selective degeneration of motor neurons and involves increased oxidative stress, overactivation of glutamate receptors and cellular calcium overload (Culmsee and Krieglstein, 2007). Ischemic neuronal death is mediated via increased calcium level, reactive oxygen species and mitochondrial dysfunction (Mattson *et al*., 2000; Wellington *et al*., 2000). In absence of trophic factors sympathetic neurons undergo cell death mediated by activation of BH3 protein resulting in inactivation of BCL2 anti-apoptotic proteins and activation of the JNK cell death pathway (Raoul *et al*.,2002; Benn and Woolf, 2004). Accumulation of misfolded proteins alters Ca^2+^ homeostasis that causes stress in ER and leads to apoptosis by activating caspases (Sánchez and Yuan, 2001; Rao *et al*.,2001; Rutkowski and Kaufman, 2004).


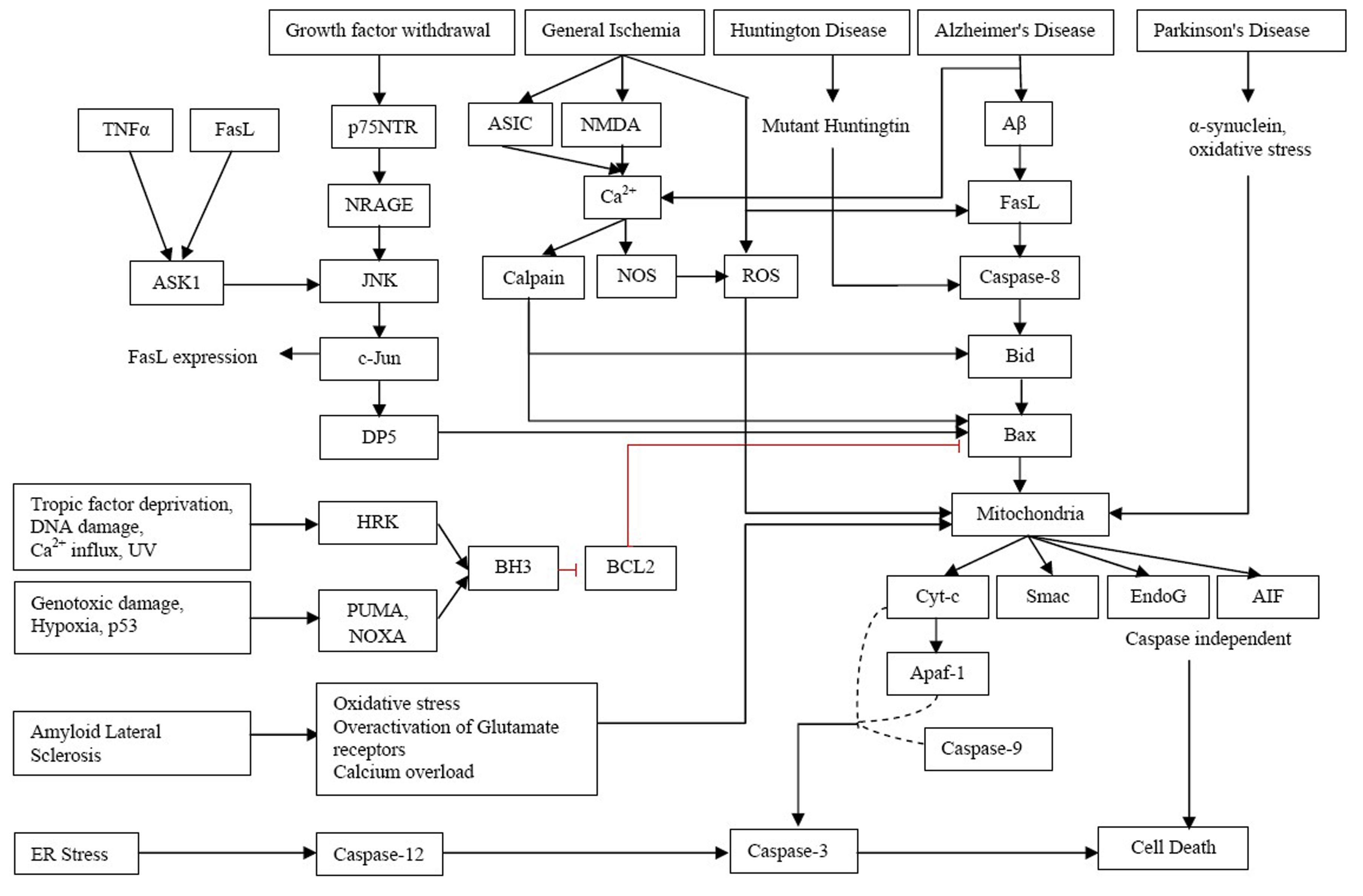


References (others refer to Manuscript references)

- Benn, S. C., andWoolf, C. J. (2004). Adult neuron survival strategies–slamming on the brakes. Nat. Rev. Neurosci. 5, 686–700. doi: 10.1038/nrn1477
- Polymeropoulos, M. H. (1998). Autosomal dominant Parkinson’s disease and alpha-synuclein. Ann. Neurol. 44, S63–S64. doi: 10.1002/ana.410440710
- Rao, R. V., Hermel, E., Castro-Obregon, S., del Rio, G., Ellerby, L. M., Ellerby, H. M., et al. (2001). Coupling endoplasmic reticulum stress to the cell death program. Mechanism of caspase activation. J. Biol. Chem. 276, 33869–33874. doi: 10.1074/jbc.M102225200
- Raoul, C., Estévez, A. G., Nishimune, H., Cleveland, D. W., deLapeyrière, O., Henderson, C. E., et al. (2002). Motoneuron death triggered by a specific pathway downstream of Fas. potentiation by ALS-linked SOD1 mutations. Neuron 35, 1067–1083. doi: 10.1016/S0896-6273(02) 00905-4
- Rutkowski, D. T., and Kaufman, R. J. (2004). A trip to the ER: coping with stress. Trends Cell Biol. 14, 20–28. doi: 10.1016/j.tcb.2003.11.001
- Sánchez, I., and Yuan, J. (2001). A convoluted way to die. Neuron 29, 563–566. doi:10.1016/S0896-6273(01)00232-X
- Su, J. H., Anderson, A. J., Cribbs, D. H., Tu, C., Tong, L., Kesslack, P., et al. (2003). Fas and Fas ligand are associated with neuritic degeneration in the AD brain and participate in beta-amyloid-induced neuronal death. Neurobiol. Dis. 12, 182–193. doi: 10.1016/S0969-9961(02)00019-0.
- Tu, H., Nelson, O., Bezprozvanny, A., Wang, Z., Lee, S. F., Hao, Y. H., et al. (2006). Presenilins form ER Ca2+ leak channels, a function disrupted by familial Alzheimer’s disease-linked mutations. Cell 126, 981–993. doi: 10.1016/j.cell.2006.06.059
- Wellington, C. L., Singaraja, R., Ellerby, L., Savill, J., Roy, S., Leavitt, B., et al. (2000). Inhibiting caspase cleavage of huntingtin reduces toxicity and aggregate formation in neuronal and nonneuronal cells. J. Biol. Chem. 275, 19831–19838. doi: 10.1074/jbc.M001475200

**Supplementary Table**

**Table S1. Boolean logical rules that govern the state of the vertices in the apoptotic network.**

| **Node** | **Boolean Rules** | **Reference** |
| --- | --- | --- |
| 1 | CASP-8 = FasL + CASP-3 – cFLIP | [1,2] |
| 2 | CASP-9 = (CASP-3 OR (CYT-C AND APAF-1)) – IAP | [3,4,25] |
| 3 | CASP-3 = (CASP-8 OR (CASP-9 AND APAF-1 AND CYT-C)) – IAP | [5,6] |
| 4 | BID = CASP-8 | [7] |
| 5 | BAX = BID – BCL2 + p53^*^ | [26,8] |
| 6 | CYT-C = BAX | [9] |
| 7 | SMAC = BAX | [10] |
| 8 | APAF-1 = CYT-C | [11] |
| 9 | IAP = – SMAC + NFκB – CASP-3^*^ | [12,13,4] |
| 10 | AKT = GF | [14] |
| 11 | BAD = NOT(AKT) | [15] |
| 12 | BCL2 = NOT(BAD) – p53^*^ | [15,16] |
| 13 | IKK = AKT | [17] |
| 14 | IκB = NOT(IKK) | [17] |
| 15 | NFκB = NOT(IκB) | [17] |
| 16 | cFLIP = NFκB | [18] |
| 17 | MDM2 = AKT OR p53 | [19] |
| 18 | p53 = DNA damage – (MDM2 AND p53^*^) | [19,20] |
| 19 | DNA damage = – p53+ CASP-3^*^ + CYT-C^*^ | [21,22,26] |
| 20 | FasL = [1,0] | Input |
| 21 | GF = [1,0] | Input |
|  | **Readout vertices** |  |
| 22 | DNA Repair = p53 | [23] |
| 23 | APOPTOSIS = DNA damage^*^ | [24] |
|  |  |  |
|  | **Threshold activation** |  |
|  | p53^*^ = p53(*t*) AND p53(*t*-1) | |
|  | CASP3^*^ = CASP-3(*t*) AND CASP-3(*t*-1) | |
|  | CYT-C^*^ = CYT-C(*t*) AND CYT-C(*t*-1) | |
|  | DNA damage^*^ = DNA damage (*t*+1) AND DNA damage (*t*) AND DNA damage (*t*-1) | |

**References**

1. Juo, P., Woo, M.S., Kuo, C.J., Signorelli, P., Biemann, H.P., Hannun, Y.A., Blenis, J. (1999) FADD is required for multiple signaling events downstream of the receptor Fas. *Cell Growth Differ* 10:797–804.
2. Krueger, A., Schmitz, I., Baumann, S., Krammer, P.H., Kirchhoff, S. (2001) Cellular FLICE-inhibitory protein splice variants inhibit different steps of caspase-8 activation at the CD95 death-inducing signaling complex. *J Biol Chem* 276:20633–20640. doi: 10.1074/jbc.M101780200.
3. Bratton, S.B., Walker, G., Srinivasula, S.M., Sun, X.M., Butterworth, M., Alnemri, E.S., Cohen, G.M. (2001) Recruitment, activation and retention of caspases-9 and -3 by Apaf-1 apoptosome and associated XIAP complexes. *EMBO J* 20:998–1009. doi: 10.1093/emboj/20.5.998.
4. Rehm, M., Huber, H.J., Dussmann, H., Prehn, J.H.M. (2006) Systems analysis of effector caspase activation and its control by X-linked inhibitor of apoptosis protein. *EMBO J* 25:4338–4349. doi: 10.1038/sj.emboj.7601295.
5. Li, P., Nijhawan, D., Budihardjo, I., Srinivasula, S.M., Ahmad, M., Alnemri, E.S., Wang, X. (1997) Cytochrome c and dATP-dependent formation of Apaf-1/caspase-9 complex initiates an apoptotic protease cascade. *Cell* 91:479–489.
6. Stennicke, H.R., Jürgensmeier, J.M., Shin, H., Deveraux, Q., Wolf, B.B., Yang, X., Zhou, Q., Ellerby, H.M., Ellerby, L.M., Bredesen, D., Green, D.R., Reed, J.C., Froelich, C.J., Salvesen, G.S. (1998) Pro-caspase-3 is a major physiologic target of caspase-8. *J Biol Chem* 273:27084–27090
7. Li, H., Zhu, H., Xu, C.J., Yuan, J. (1998) Cleavage of BID by caspase 8 mediates the mitochondrial damage in the Fas pathway of apoptosis. *Cell* 94:491–501.
8. Desagher S, Osen-Sand A, Nichols A, Eskes R, Montessuit S, Lauper S, Maundrell K, Antonsson B, Martinou JC (1999) Bid-induced conformational change of Bax is responsible for mitochondrial cytochrome c release during apoptosis. *J Cell Biol* 144:891–901.
9. Zamzami N, Kroemer G (2001) The mitochondrion in apoptosis: how Pandora’s box opens. *Nat Rev Mol Cell Biol* 2:67–71. doi: 10.1038/35048073
10. Bernardi P, Scorrano L, Colonna R, Petronilli V, Di Lisa F (1999) Mitochondria and cell death. Mechanistic aspects and methodological issues. *Eur J Biochem* 264:687–701.
11. Shiozaki EN, Chai J, Shi Y (2002) Oligomerization and activation of caspase-9, induced by Apaf-1 CARD. *Proc Natl Acad Sci USA* 99:4197–4202. doi: 10.1073/pnas.072544399
12. Datta R, Oki E, Endo K, Biedermann V, Ren J, Kufe D (2000) XIAP regulates DNA damage-induced apoptosis downstream of caspase-9 cleavage. *J Biol Chem* 275:31733–31738. doi: 10.1074/jbc.M910231199
13. Du C, Fang M, Li Y, Li L, Wang X (2000) Smac, a mitochondrial protein that promotes cytochrome c-dependent caspase activation by eliminating IAP inhibition. *Cell* 102:33–42.
14. Brunet A, Datta SR, Greenberg ME (2001) Transcription-dependent and -independent control of neuronal survival by the PI3K-Akt signaling pathway. *Curr Opin Neurobiol* 11:297–305.
15. Datta SR, Dudek H, Tao X, Masters S, Fu H, Gotoh Y, Greenberg ME (1997) Akt phosphorylation of BAD couples survival signals to the cell-intrinsic death machinery. *Cell* 91:231–241.
16. Haldar S, Negrini M, Monne M, Sabbioni S, Croce CM (1994) Down-regulation of bcl-2 by p53 in breast cancer cells. *Cancer Res* 54:2095–2097.
17. Karin M (1999) The beginning of the end: IkappaB kinase (IKK) and NF-kappaB activation. *J Biol Chem* 274:27339–27342.
18. Micheau O, Lens S, Gaide O, Alevizopoulos K, Tschopp J (2001) NF-kappaB signals induce the expression of c-FLIP. *Mol Cell Biol* 21:5299–5305. doi: 10.1128/MCB.21.16.5299-5305.2001
19. Lee M-H, Lozano G (2006) Regulation of the p53-MDM2 pathway by 14-3-3 sigma and other proteins. *Semin Cancer Biol* 16:225–234. doi: 10.1016/j.semcancer.2006.03.009
20. Bates S, Vousden KH (1996) p53 in signaling checkpoint arrest or apoptosis. *Curr Opin Genet Dev* 6:12–18.
21. Canman CE, Lim DS, Cimprich KA, Taya Y, Tamai K, Sakaguchi K, Appella E, Kastan MB, Siliciano JD (1998) Activation of the ATM kinase by ionizing radiation and phosphorylation of p53. *Science* 281:1677–1679.
22. Kastan MB, Lim DS (2000) The many substrates and functions of ATM. *Nat Rev Mol Cell Biol* 1:179–186. doi: 10.1038/35043058
23. Jõers A, Jaks V, Kase J, Maimets T (2004) p53-dependent transcription can exhibit both on/off and graded response after genotoxic stress. *Oncogene* 23:6175–6185. doi: 10.1038/sj.onc.1207864
24. Roos WP, Kaina B (2006) DNA damage-induced cell death by apoptosis. Trends Mol Med 12:440–450. doi: 10.1016/j.molmed.2006.07.007.
25. Mai, Z., Liu, H. (2009) Boolean network-based analysis of the apoptosis network: irreversible apoptosis and stable surviving. *J Theor Biol* 259:760–769. doi: 10.1016/j.jtbi.2009.04.024.
26. Karpinich, N.O., Tafani, M., Rothman, R.J., Russo, M.A., Farber, J.L. (2002) The course of etoposide-induced apoptosis from damage to DNA and p53 activation to mitochondrial release of cytochrome c. *J Biol Chem* 277:16547–16552. doi: 10.1074/jbc.M110629200
